# RhoGTPase signalling at epithelial tight junctions: Bridging the GAP between polarity and cancer

**DOI:** 10.1016/j.biocel.2015.02.020

**Published:** 2015-07

**Authors:** Ceniz Zihni, Stephen James Terry

**Affiliations:** a UCL Institute of Ophthalmology, University College London, 11-43 Bath Street, London EC1 V 9EL, UK; b Randall Division of Cell & Molecular Biophysics, New Hunt's House, Guy's Campus, Kings College London, London SE11UL, UK

**Keywords:** RhoGTPase, GEF, GAP, Signalling, Cancer

## Abstract

The establishment and maintenance of epithelial polarity must be correctly controlled for normal development and homeostasis. Tight junctions (TJ) in vertebrates define apical and basolateral membrane domains in polarized epithelia via bi-directional, complex signalling pathways between TJ themselves and the cytoskeleton they are associated with. RhoGTPases are central to these processes and evidence suggests that their regulation is coordinated by interactions between GEFs and GAPs with junctional, cytoplasmic adapter proteins. In this InFocus review we determine that the expression, localization or stability of a variety of these adaptor proteins is altered in various cancers, potentially representing an important mechanistic link between loss of polarity and cancer. We focus here, on two well characterized RhoGTPases Cdc42 and RhoA who's GEFs and GAPs are predominantly localized to TJ via cytoplasmic adaptor proteins.

## Signalling network facts

• RhoGTPases are molecular switches that control TJ assembly and function and are regulated by guanine nucleotide exchange factors (GEF) and GTPase-activating proteins (GAP) that promote a cycling between a GTP-bound, active, and a GDP-bound, in-active state.
• Tight junction cytoplasmic ‘plaque’ proteins, that include scaffold/adaptor proteins, attach the cytoskeleton to the junctional membrane and act as a platform for a variety of signalling proteins.
• Tight junction cytoplasmic plaque adaptor proteins act as targeting sites for different Cdc42 and RhoA GEFs and GAPs that contain a variety of targeting domains including (Bin Amphiphysin-Rvs) BAR, pleckstrin homology (PH), Src Homology 3, (SH3), (PSD-95/Dlg/ZO-1) PDZ domains to modulate correct spatio-temporal regulation of RhoGTPases during TJ assembly and function.
• Various junctional cytoplasmic adaptor proteins are often dysregulated in cancer by degradation, dissociation from tight junctions, altered expression or subjected to pro-EMT transcriptional repression thereby, providing a potentially important mechanistic link between loss of polarity and cancer.

## 1 Introduction

Epithelia provide functional barriers that separate our organs and tissues from the external environment by forming intercellular junctions that adhere cells tightly to each other. In vertebrates tight junctions, a component of the apical junctional complex (APC) that also include more basally localized adherens junctions (AJ), regulate cell-cell adhesion, paracellular permeability and maintain epithelial cell surface polarity by forming a discreet boarder between apical and basolateral cell surface domains (Zihni et al., 2014a).

**Fig. 1:**
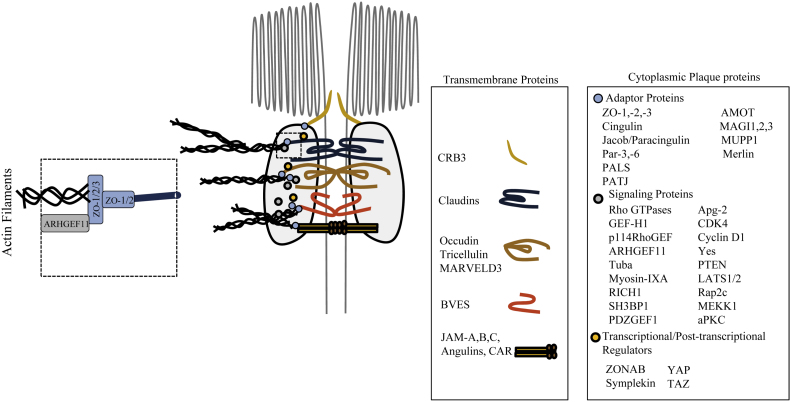
Composition of tight junctions. TJ transmembrane proteins include the tri-span BVES, the MARVEL domain-containing MARVELD3, occludin and tricellulin, the tetraspan claudin family, Crb3 (an apical determinant that forms a complex with PALS1 and PATJ), and JAMs, CAR and angulins that are immunoglobulin superfamily members. These transmembrane proteins interact with a cytoplasmic network of proteins, that form a ‘plaque’ that links the transmembrane proteins to the cytoskeleton. The cytoplasmic plaque is composed of various scaffolding proteins that contain protein–protein interaction domains and can interact directly with F-actin or microtubules acting as adaptors. This plaque also includes signalling proteins, such as GTPases (e.g. RhoGTPase and Raps) and their regulators that control junction assembly and function. Another class of plaque proteins, transcriptional and post-transcriptional regulators are anchored at the TJ, but can dissociate from these structures and enter the nucleus to regulate gene expression. Note that the proteins indicated here are major examples of TJ components, but do not represent a complete list of proteins that can be found at TJ. Additionally, the figure does not represent exclusivity, in other words some proteins e.g. Jacob and SH3BP1, Cdc42 and RhoA are not only restricted to TJ.

Tight junctions are composed of 2 groups of proteins: transmembrane proteins that mediate cell-cell adhesion, and cytoplasmic ‘plaque’ proteins that attach the cytoskeleton to the junctional membrane and act as a platform for a variety of signalling proteins (Zihni et al., 2014) (Fig. 1). Despite being anchored to the cytoskeleton TJs are not static structures but dynamic and require complex remodelling mediated by the coordinated activities of vesicle trafficking machineries and the actin and microtubule cytoskeletons, during their assembly/disassembly, function and maintenance (Ivanov et al., 2005; Ivanov 2008; Rodgers and Fanning, 2011).

## 2 Functions, cascades, and key molecules

### 2.1 TJ adaptor protein-associated GEFs and GAPs as key regulators of RhoA and Cdc42

RhoGTPases, members of the Ras superfamily, serve as molecular switches that control TJ assembly and function. Guanine nucleotide exchange factors (GEF) and GTPase-activating proteins (GAP) promote a cycling of the RhoGTPase between a GTP-bound, active state, and a GDP-bound, in-active state. In the GTP on state they can activate downstream effector proteins, thus they are able to transmit and convert signals from adhesion molecules, growth factors and cytokines into biological effects to control diverse cellular processes such as regulation of actin polymerization, organization and mechanics, vesicle trafficking and gene expression, to coordinate the formation, dissolution and regulation of junctions. Accumulating evidence suggest various adapter protein constituents of the TJ cytoplasmic plaque, act as targeting or ‘docking’ sites for GEFs and GAPs, facilitating spatio-temporal regulation of RhoGTPases (Table 1 and Fig. 1). Junctional adaptor proteins not only localize GEFs and GAPs to regulate junction assembly and function, but the status of cell-cell contact and degree of junctional maturity and thus polarity dictates recruitment of specific GEFs and GAPs at specific times. The outline of this principle feature of polarity was first proposed some years ago (Drubin and Nelson, 1996), but a great body of work since has enabled us to begin to understand the underlying molecular principles of this mechanism.

### 2.2 Spatial/temporal control of RhoGTPases during TJ assembly, maturation and apical specification

In many epithelia, Cdc42-driven filopodia mediated induction of cell-cell contact initiates junction assembly; although a Rac/lamellapodia-dependent mechanism also exists depending on the epithelial cell-type. When epithelial cells contact each other, E-cadherin, a transmembrane protein of AJ, is recruited to sites of cell-cell contact at the tips of filopodia by a mechanism that is thought to involve interaction of the cytoplasmic domain of E-cadherin with C3G, a GEF for Rap1, promoting Cdc42 activation and directional vesicle transport of E-cadherin (Takeichi 2014). Engagement of opposing E-cadherin molecules results in the formation of a cluster, followed by the assembly of additional adjacent puncta, generating a zipper-like structure that develops into mature, linear cell-cell contacts. Recently, E-cadherin engagement was shown to be linked to c-src-mediated phosphorylation of cortactin, by receptor protein tyrosine phosphatase (RPTPa) (Truffi et al., 2014). Conversely, E-cadherin expression is modulated in order to reduce adhesion during epithelial-mesenchymal transition (EMT) a process that results in migration and invasion during normal development and cancer (Gheldof and Berx, 2013).

**Fig. 2:**
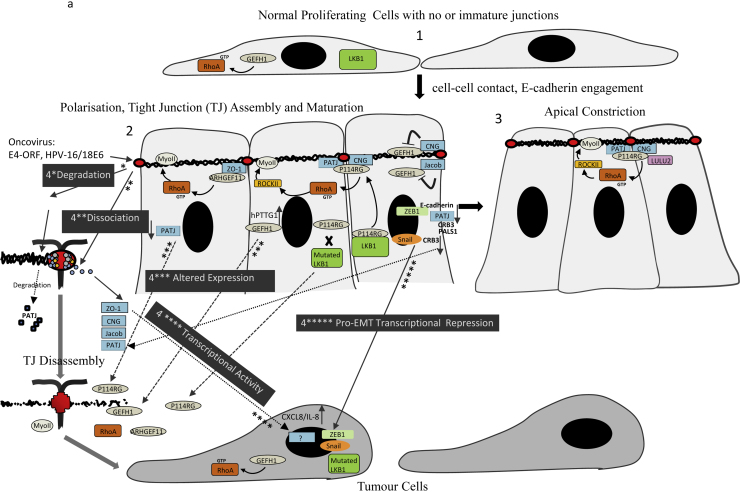

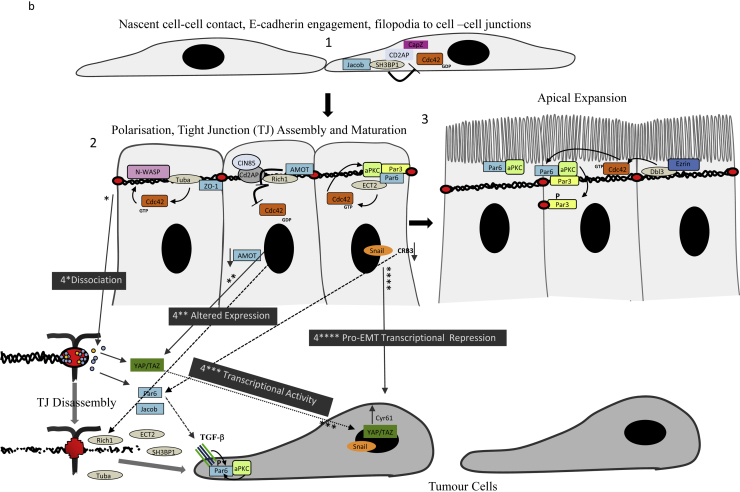
Tight junction adaptor proteins and their role in the regulation of RhoA and Cdc42. (a) (1) At low-density cells lack mature junctions and undergo proliferation. The RhoA activator GEF-H1 is distributed throughout the cytoplasm promoting the formation of stress fibres and cell proliferation. (2) During junction maturation, GEF-H1 is sequestered by adaptors cingulin (CNG) and JACOP/paracingulin, resulting in its inactivation and downregulation of cytoplasmic RhoA when epithelial cells reach confluence and are no longer proliferating. Conversely, junction assembly does require restricted RhoA activity promoted by ARHGEF 11 and p114RhoGEF recruited by ZO-1 (initially at primordial junctions) and cingulin/PATJ respectively. Recently, some activators of these GEFs have been identified for instance, LKB1 and LULU2 activates p114RhoGEF during junction assembly (2) and apical constriction (3), respectively. (4) In various cancers junctional adaptor proteins are either * degraded by onco-viruses e.g. PATJ, ** dissociate directly e.g. ZO-1, or indirectly e.g. cingulin/Jacob requires ZO-1 for normal junctional localization, *** altered expression of adaptors e.g. decrease in PATJ expression leading to defects in TJ junction assembly or increase in GEF-H1 expression leading to pro-proliferative activity of RhoA, **** increased transcriptional activity e.g. dissociated ZO-1, from TJ, promotes increase transcription of CXCL/IL-8, *****EMT, e.g. ZEB1-dependent down regulation of E-cadherin and Crb3 complex including PATJ and Snail-dependent down regulation of Crb3 that results in decreased localization of PATJ at the tight junction. In addition to the loss of junctional adaptors Cingulin and PATJ, p114RhoGEF function may be impaired by loss of LKB1 function. (b) (1) In many types of epithelial cells junction assembly is initiated by filopodia-mediated cell-cell contact, and E-cadherin engagement. Local inactivation of Cdc42 by the GAP SH3BP1 that is recruited at nascent cell-cell contacts by Jacob, is required to convert filopodia to mature junctions. (2) The Cdc42 GEF ECT2 is suggested, at least in a cell specific manner, to be recruited by the Par complex to regulate junction assembly and a second Cdc42 GAP RICH1, is recruited by the adapter AMOT after junction assembly to regulate Cdc42 activity, junctional maturation and integrity. Evidence suggests that RICH1 which interacts with endocytic factors Cd2AP and CIN85 may modulate junctional integrity via regulating vesicle trafficking of TJ components. (3) The Cdc42 GEF Dbl3 also functions after junction assembly to drive Par polarity signalling, apical expansion and brush border induction. (4) In various cancers junctional adaptor proteins either * dissociate directly e.g. ZO-1 which evidence suggest would affect correct localization of Jacob and thus SH3BP1,**altered expression e.g. reduced expression of AMOT would result in a reduction of sequestration of RICH1 and also the transcriptional co-activators YAP/TAZ at junctions, *** transcriptional activity e.g. nuclear translocation of YAP/TAZ promotes increased transcription of the growth factor Cyr61, **** EMT, e.g. reduction of Crb3 expression by Snail results in loss of Par6 from junctions which has been proposed to localize in cancer cells to the TGF-β receptor and confer a tumorigenic role.

The mechanism by which filopodia is converted to maturing junctions, following nascent cell-cell contact, involves, at least in part, the GAP SH3BP1 which forms a complex with both a junctional adaptor protein Jacob/paracingulin and the scaffold protein CD2AP via its N-terminal Bar domain and C-terminal SH3B domains, respectively (Table 1 and Fig. 2b) (Elbediwy et al., 2012). Not only does this complex attenuate actin-driven morphogenic processes by locally inhibiting Cdc42 signalling, Capz also forms a complex with SH3BP1, indicating a direct regulation of actin polymerization. Recently, a mechanism for the activation of SH3BP1 GAP activity was proposed, involving its dissociation from the PlexinD1 receptor (Tata et al., 2014). SH3BP1 interacts with Plexin D1 via its BAR domain and dissociates from this receptor following an external stimulus involving Sema3E interaction with Plexin D1, to promote regulation of Rac1. Whether this mechanism is associated with activation of SH3BP1 during nascent cell-cell contacts in order to regulate Cdc42 remains to be determined. In addition to transient inhibition of Cdc42, during nascent cell-cell contact, cadherin engagement promotes recruitment of p190RhoGAP/ARHGAP35 to AJ promoting RhoA inactivation (Zihni et al., 2014a). TJ formation, induced by the junctional membrane protein BVES, then promotes recruitment of RhoA GEF, GEF-H1 by junctional adaptors cingulin and/or JACOB/paracingulin (Fig. 2a). The PH domain of GEF-H1 was found to interact with the rod domain of cingulin resulting in sequestration and inhibition of GEF-H1 at TJ (Aijaz et al., 2005; Benais-Pont et al., 2003; Guillemot et al., 2008; Russ et al., 2011). A loss of GEF-H1 at TJ, resulting in its activation promotes RhoA-dependent gene expression, cell cycle progression, cell spreading and migration (Zihni et al., 2014a). Additionally, GEF-H1 dissociation and activation of RhoA/ROCKII, in a calcium depletion model, promoted junction disassembly (Samarin et al., 2007). Studies using non-polarized cells have shown that GEF-H1 is also sequestered by microtubules (MT) and its activity is inhibited by phosphorylation at Ser 885. Thrombin and lysophosphatidic acid (LPA) stimulation of GPCR promotes displacement from MTs and thrombin/PP2A dependent de-phosphorylation resulting in its activation (Meiri et al., 2014). It is unclear whether TJ and MT dependent inhibitory sequestration of GEF-H1 represent interdependent mechanisms in polarizing cells, although a recent study suggests that cinguin links TJs to MTs (Yano et al., 2013).

Despite the inhibition of GEF-H1 as nascent cell-cell contacts mature, junctional assembly and maturation further require localized RhoA activation at cell-cell contacts (Fig. 2a). Firstly, the RhoA GEF ARHGEF11 localizes initially to primordial AJ, then to TJ, interacting with ZO-1 via its C-terminus, in polarized epithelial cells to regulate the circumferential actomyosin belt (Itoh et al., 2012), supporting a model whereby RhoA signalling at TJs is also affected by AJs, during junction formation (McCormack et al., 2013). A second RhoA GEF p114 RhoGEF localizes to TJs due to interactions with cingulin and PALS1-associated tight junction protein (PATJ) via its N-terminal pleckstrin homology (PH) domain and C-terminal PDZ (PSD-95/Dlg/ZO-1) binding motif, respectively (Terry et al., 2011; Nakajima and Tanoue, 2012). The Ser/Thr kinase LKB1, an important polarity factor in metazoans, activates p114RhoGEF/RhoA, which is required for junction assembly (Xu et al., 2013). Active P114RhoGEF forms a complex with cingulin, MyosinIIA and RockII to regulate tension of the circumferential actomyosin belt that is required for TJ assembly and morphogenesis (Terry et al., 2011). The GEF Ect2 has been shown to regulate Cdc42 by interacting with the junctional adaptor Par6, of the Par6-aPKC-Par3 complex, via its N-terminal and C-terminal regions, and associate at TJs in Madin–DarbyCanineKidney (MDCK) cells (Fig. 2b) (Zihni et al., 2014a). Binding of active Cdc42 to Par6, its effector, promotes activation of aPKC which various studies indicate, is required for TJ formation, although Ect2 RhoGTPase specificity may depend on the type of epithelial cell (Zihni et al., 2014a). A second structurally related Cdc42 GAP to SH3BP1, RICH1, is required for junctional maturation and maintenance, which is recruited to TJs via the junctional adaptor protein angiomotin (AMOT), via its N-terminal BAR domain, that regulates its GAP activity (Fig. 2b) (Wells et al., 2006). Both Rich1 and AMOT contain BAR domains that are crescent-shaped, sensing membrane curvature, capable of producing membrane bending and tubulation and are often involved in the formation of vesicles. Rich1 interacts with CIN85 and CD2AP, both important proteins in endocytic regulation of EGF and HGF receptors and knock down of Rich1 results in a loss of integrity of junctions, and distribution of TJ components into circular structures and fragmented spots within the cell (Wells et al., 2006). Thus, Rich1/AMOT regulated Cdc42, at junctions may affect TJ integrity via regulating internalization of components of the TJ, further supporting a major role for Cdc42 in vesicle trafficking during epithelial polarization and TJ function (Harris and Tepass, 2010). It is intriguing therefore, to speculate whether the Cdc42 GAP that precedes Rich1, SH3BP1 that also contains an N-terminal BAR domain plays a role in vesicle trafficking, during TJ assembly.

Other GEFs and GAPs that function after junctional assembly include the Cdc42 GEF Tuba that regulates tension and configuration of junctions (Fig. 2b) (Otani et al., 2006; Oda et al., 2014). Additionally, evidence suggest that junction assembly and maturation culminates with RhoA and Cdc42 driving opposing processes where the GEF Dbl3 drives expansion of the apical domain and brush border induction (Fig. 2b) (Zihni et al., 2014b) and activation of p114RhGEF by Lulu2 drives RhoA dependent constriction of the circumferential actomyosin belt, inducing apical constriction (Fig. 2a) (Nakajima and Tanoue, 2012).

## 3 Associated pathologies and therapeutic implications-focus on cancer

The development of cancer is often associated with a loss of cohesion between epithelial cells and altered expression and/or distribution of TJ proteins, accompanied by a loss of apicobasal polarity (Martin, 2014; Lo et al., 2012). Changes in the expression levels and/or localization of various TJ cytoplasmic plaque adapter proteins have been reported in various cancers. Such a dysregulation would directly affect correct recruitment of junctional GEFs and GAPs and therefore RhoGTPase signalling, and thus disrupt TJ assembly, maturation, and integrity (Fig. 2a and b). Furthermore, evidence suggests that, at least, in some cases loss of junctional cytoplasmic plaque proteins during cancer is accompanied by regulation of tumour promoting genes. For example ZO-1 which is required for the RhoA GEF ARHGEF11 and Cdc42 GEF Tuba to localize and function at TJ (Table 1 and Fig. 2a and b) relocalizes from TJs in several breast cancer cell-lines and regulates CXCL8/IL-8 expression, a protein strongly implicated in tumour invasion (Brysse et al., 2011). Cingulin and paracingulin, targeting sites for p114RhoGEF and GEF-H1 RhoA GEFs and Cdc42 GAP SH3BP1, interact with ZO-1 in vitro through their N-terminal ZIM motif and require ZO-1 for normal recruitment to TJs (Pulimeno et al., 2011). Thus, this work strongly suggests that loss of ZO-1 in cancer is likely to affect cingulin/paracingulin localization (Fig. 2a and b).

AMOT expression, required for TJ localization of the Cdc42 GAP RICH1 (Table 1 and Fig. 2b), is deceased in lung cancer, resulting in increased nuclear translocation of YAP/TAZ, oncogenic transcriptional co-activators that are otherwise thought to be sequestered/inhibited at TJ, and increased expression of the growth factor Cyr61 (Hsu et al., 2014) (Fig. 2b). Crb3 is considered as a cancer suppressor and widely expressed in epithelial cells (Li et al., 2015). In mouse kidney epithelial cells repression of Crb3 expression correlates with increased tumorigenic potential. Evidence suggests that Crb3 complex components, including PALS-1 and PAT-J (the p114RhoGEF junctional adaptor), are both direct targets and regulators of EMT transcriptional repressors (Li et al., 2015). In colorectal carcinoma cells SW480 and undifferentiated breast tumour cells MDA-MB-231 PATJ, in addition to Crb3 and PALS1 are up-regulated after the E-cadherin repressor ZEB1 is silenced. The transcription of Crb3 is repressed by the transcriptional repressor Snail thereby abolishing the localization of the Crb complex at TJs in MDCK cells (Fig. 2a). In TGF-β-treated mouse mammary epithelial Eph4 cells grown at high density, Crb3 knockdown increases the expression of Snail and predisposed cells to EMT. PAT-J is also targeted for degradation by viral oncoproteins E4-ORF, HPV-16 E6 and HPV-18 E6 (Fig. 2a) (Lo et al., 2012). In addition to the Crb3 complex Snail also abolishes TJ localization of the Par complex in MDCK cells (Fig. 2b) (Li et al., 2015). Phosphorylation of Par6, by the TGF-β receptor and aPKC, has been correlated with reduced survival in breast cancer patients, being associated with more invasive tumours and also increased migration of non-small-cell lung cancer (NSCLC) cells (Fig. 2b) (Viloria-Petita et al., 2009; Gunaratne et al., 2013). Par6 is a junctional adaptor protein that is reported to be a target for ECT2 and activation of Cdc42-dependent Par6-aPKC/Par3 is required for junction assembly. Thus collectively, these studies indicate that a loss of junctional localization of this adaptor promotes a switch from a Cdc42-dependent pro junction assembly role to a tumorigenic role via relocalization to a distinct functional membrane complex (Fig. 2b). In addition to a potential loss of inhibitory sequestration of GEFH1 at TJ, increased expression of human pituitary tumour-transforming gene 1 (hPTTG1), a transcription factor overexpressed in many types of tumour, transcriptionally activates GEFH1 expression in a highly metastatic breast cancer cell-line (Liao et al., 2012) which may overcome the inhibitory capacity of TJ-bound cingulin (Fig. 2a).

## 4 Concluding remarks

As we continue to understand the structure and molecular mechanisms of TJ regulation, it is becoming clear that this complex structure and associated signalling modules, is potentially an important therapeutic target for anticancer research. Presently, a focus on drugs being developed, include those that manipulate the permeability regulation properties of transmembrane claudins to interfere with the path of tumour metastasis (Martin, 2014). Understanding further the mechanism and functions of other TJ molecules including cytoplasmic plaque protein adaptor molecules and interdependent RhoGTPase signalling networks, as highlighted in this review, will enable further possibilities to utilize/manipulate these TJ components and related-signalling networks as targets or strategies for cancer therapy during earlier stages of cancer development. Such an example may be illustrated by the recent discovery of LKB1, inactivated in a variety of cancers, as an activator of p114RhoGEF-driven junction assembly, which occurs via recruitment by cingulin and PATJ junctional adaptors. LKB1 possess a unique feature as a tumour suppressor by promoting cell survival via suppressing cell proliferation under energetic stress conditions (Zhou et al., 2014). Thus, Since, LKB1-null cells ability to sense energetic stress is impaired and are therefore more vulnerable, the potential to utilize drugs such as phenformin in order to kill cells when LKB1 function is absent, has arisen.

## Figures and Tables

**Table 1:** Tight junction targeting domains of Cdc42/RhoA GEFs and GAPs.

	Targeting sequence/domain	Junctional component	Function	Reference(s)
**GEF**				
Tuba	C-terminal region containing SH3 domains, plus additional, undetermined aa residues	TJ ZO-1 (CPAP), Tricellulin/MARVELD2 (ITMP)	TJ configuration, tension via activation of Cdc42/N-WASP Effector, promoting actin polymerization	Otani et al. (2006), Oda et al. (2014)
DBL3	N-terminal Sec 14-like region, pleckstrin homology (PH) domain	Ezrin (TJAMP), membrane phospholipids?	TJ positioning via activation of Cdc42/Par6-aPKC Effector complex	Ognibene et al. (2011), Zihni et al. (2014b)
ECT2	N-terminal regulatory domain (cyclin b6, BRCT1/2, S), C-terminal (DH, PH) region	TJ Par6 (CPAP)	TJ assembly and/or maintenance via activation of the Cdc42/Par6-aPKC-Par3 effector complex	Liu et al. (2004, 2006)
p114 RhoGEF	N-terminal pleckstrin homology (PH) domain, C-terminal PDZ (PSD-95/Dlg/ZO-1) binding motif (PBM)	TJ Cingulin (CPAP), TJ PALS 1-associated tight junction Protein (PATJ) (CPAP)	TJ assembly and maturation via RhoA/ROCKII-dependent MLC phosphorylation	Terry et al. (2011), Nakajima and Tanoue (2012)
ARHGEF11	C-terminal region	TJ ZO-1 (CPAP)	TJ assembly and maturation via RhoA-dependent MLC phosphorylation	Itoh et al. (2012)
GEFH1	PH domain	TJ Cingulin/JACOB (CPAP)	Inhibition of Rho A dependent proliferation following TJ assembly	Aijaz et al. (2005)

**GAP**				
SH3BP1	N-terminal Bar domain C-terminal SH3 domain	TJ Jacob (paracingulin) (CPAP) CD2AP	TJ assembly via regulation of Cdc42 at nascent cell-cell contacts, inactivation of actin Polymerization and Actin capping	Elbediwy et al. (2012)
Rich1	N-terminal Bar domain	TJ angiomoitin (AMOT) (CPAP)	TJ maturation/integrity via regulation of Cdc42, linking TJ to intracellular protein trafficking	Wells et al. (2006)

CPAP, cytoplasmic plaque adapter protein; ITMP, integral transmembrane protein; TJAMP, tight junction apical margin protein.
